# Genome Sequences of *Brucella melitensis*, Isolated from Blood Samples of Brucellosis Patients in Malaysia

**DOI:** 10.1128/genomeA.00689-17

**Published:** 2017-08-03

**Authors:** Jama’ayah Mohamed Zahidi, Norazah Ahmad, Bee Yong Tay, Rohaidah Hashim, Evie Khoo, Nurizan Ahmad, Chung Yan Yee, Nur Qhaledaziea Dolhan

**Affiliations:** a Bacteriology Unit, Infectious Disease Research Centre, Institute for Medical Research, Kuala Lumpur, Malaysia; b Veterinary Research Institute, Ipoh, Malaysia

## Abstract

Human brucellosis is a neglected zoonotic disease and has widespread geographical distribution. *Brucella melitensis* has caused outbreaks and sporadic cases in Malaysia. Here, we present the whole-genome sequences of four *B. melitensis* strains isolated from brucellosis patients in Malaysia.

## GENOME ANNOUNCEMENT

Brucellosis is a highly infectious zoonotic disease affecting humans and diverse groups of wild and domestic animals, with more than 500,000 human cases reported annually worldwide (1, 2). The disease, caused by *Brucella* spp., is mostly transmitted to humans through direct contact or by ingestion of unpasteurized infected dairy products (3, 4). Among the six classically known *Brucella* spp., *B. melitensis* is the most pathogenic species infecting humans (5). It is also the most frequent *Brucella* sp. isolated from blood cultures of humans infected with brucellosis. The genetic characterization of *B. melitensis* isolates is important for epidemiological trace-back investigations of both outbreak and sporadic brucellosis. Multiple-locus variable-number tandem-repeat analysis is generally used for genotyping *Brucella* isolates, but assays are laborious, expensive, and time-consuming.

In order to identify the genetic characteristics of strains from Malaysia, we present here the genome sequences of four *B. melitensis* strains isolated from human patients. The four strains were isolated from blood samples of brucellosis patients who resided in different districts of Johor State (located south of Peninsular Malaysia) in 2015. Strains BMM 13/15 and BMM 26/15 were isolated from Batu Pahat, Johor, while strains BMM 15/15 and BMM 19/15 were isolated from Johor Bahru and Muar, respectively. These strains were inoculated onto brucella agar and incubated at 37°C for 48 h. The genomic DNA was extracted using a MasterPure DNA purification kit (Epicentre, Illumina, USA). Then, DNA libraries were prepared using a Nextera XT DNA library preparation kit (Illumina). Genomic DNA samples of the four clinical strains of *B. melitensis* were sequenced on the Illumina MiSeq platform using a paired-end (2 × 251-bp) sequencing protocol. The generated sequencing reads were filtered by removing low-quality sequences with the BBDuk version 36 toolkit (http://jgi.doe.gov/data-and-tool/bbtools). The high-quality sequence read data were successfully assembled *de novo* into contigs by using SPAdes version 3.9.0 (6) software.

The draft assemblies of the four clinical strains were annotated using the NCBI Prokaryotic Genome Annotation Pipeline version 4.1. Using Bowtie2 version 2.3, we determined that the sequences shared high nucleotide identity (>99%) with the reference genomes of *B. melitensis* strain 16M (GenBank accession no. NC_003317 and NC_003318) (7). Using these mappings to the reference genomes, we determined the number of single nucleotide polymorphisms (SNPs) by using the SnpEff tool version 4.3i (8). A range of 2,540 to 2,548 SNPs was identified in the four clinical genome sequences (Table 1). This implied that the clinical strains of *B. melitensis* are different from the reference strain. All assemblies contained 3 rRNAs and have a 57.2% G+C content. The other genome assembly metrics (i.e., numbers of contigs, assembly size, *N*_50_, average coverage, and number of genes, tRNAs, and SNPs) are provided in Table 1. Further detailed comparative analysis of genetic polymorphisms will help in understanding the expression of certain genes, which can improve epidemiological typing tools and preventive strategies to control brucellosis.

**TABLE 1:** Summary metrics of whole-genome assemblies of four clinical *B. melitensis* strains isolated from Johor, Malaysia

Strain	No. of contigs	Assembly size (bp)	*N*_50_ (bp)	Average coverage (×)	No. of genes	No. of tRNAs	No. of SNPs	Accession no.
BMM 13/15	35	3,288,783	189,507	64.55	3,359	49	2,545	NCRL00000000
BMM 15/15	44	3,288,964	176,854	39.36	3,366	49	2,540	NCRJ00000000
BMM 19/15	46	3,288,126	140,693	38.17	3,367	49	2,546	NCRI00000000
BMM 26/15	39	3,288,579	249,479	60.82	3,364	49	2,548	NBBM00000000

### Accession number(s).

The whole-genome shotgun sequences reported here have been deposited in GenBank under BioProject PRJNA380526 and are available under the accession numbers listed in Table 1.
